# Population attributable fraction of modifiable risk factors for Alzheimer disease: A systematic review of systematic reviews

**Published:** 2016-07-06

**Authors:** Narjes Hazar, Leila Seddigh, Zahra Rampisheh, Marzieh Nojomi

**Affiliations:** 1 Department of Community Medicine, School of Medicine, Tehran University of Medical Sciences, Tehran, Iran; 2 Department of Community Medicine, School of Medicine, Iran University of Medical Sciences, Tehran, Iran; 3 Department of Community Medicine, School of Medicine, Preventive Medicine and Public Health Research Center, Iran University of Medical Sciences, Tehran, Iran

**Keywords:** Alzheimer Disease, Risk Factors, PAF

## Abstract

**Background:** Alzheimer’s disease (AD) is the most common type of dementia. Demonstrating the modifiable risk factors of AD can help to plan for prevention of this disease. The aim of the current review was to characterize modifiable cardiovascular risk factors of AD using existing data and determine their contribution in AD development in Iran and the world.

**Methods:** The systematic search was done in Medline, Scopus, and Cochrane databases from inception to May 2014 to find systematic reviews or meta-analyses about association between AD and cardiovascular modifiable risk factors included diabetes, hypertension (HTN), physical inactivity, smoking, hypercholesterolemia, and overweight and obesity. The population attributable fraction (PAF) was calculated for these risk factors in Iran and the world.

**Results:** Of 2651 articles, 11 were eligible for data extraction after assessing relevancy and quality. Diabetes mellitus (DM) type 2, smoking, physical inactivity, overweight and obesity were significantly associated with increased risk of AD. Physical inactivity with 22.0% and smoking with 15.7% had the highest PAF for AD in Iran and the world, respectively.

**Conclusion:** Our findings demonstrated that modifiable cardiovascular risk factors could increase the risk of AD. Moreover, about one-third of AD cases were attributed to five modifiable risk factors.

## Introduction

Dementia is one of the most common and debilitating disorders in late life. The number of people affected by dementia in 2010 was estimated to be 35.6 million worldwide and 4.6 million new cases occur annually. The number doubles every 20 years.^1^

Alzheimer’s disease (AD) is the most common type of dementia that includes 60-80% of this problem. The prevalence of AD is growing mainly due to demographic changes and increasing life expectancy. Nowadays, approved drugs have little effect on dementia, particularly on AD, and are unable to stop progression of disease. Therefore, identification and implementation of preventive strategies seem to be more practical and essential to fight with this disease.^2^

Finding new ways to postpone the incidence of the disease is considered a valuable therapy too. It is generally accepted that postponing symptoms even for 5 years can cut down the disease incidence to half.^3^ In other words, if this delay extends to 1 year, it will save 9 million lives in the next 40 years.^2^

Studies that focus on risk factors are valuable among investigations of neurological diseases because they are directly associated with primary prevention.^4^

Numerous studies have demonstrated that various modifiable risk factors are associated with AD such as obesity,^5^ smoking, diabetes, hypertension (HTN) in midlife,^6^^,^^7^ and hypercholesterolemia.^1^^,^^8^^-^^10^ It is also verified that physical activity would be a protective factor against AD.^2^^,^^4^^,^^9^

Barnes and Yaffe^2^ have focused on studying about the contribution of some modifiable risk factors on AD incidence and have found that nearly half of AD could be attributed to these factors. It has been shown with 10-25% reduction of the prevalence of all these risk factors, almost 1.1-3 million new cases would be averted, respectively.

Since Iranian population are undergoing the aging phenomena, which can be accompanied by ‎increasing the incidence of AD,4 we decided to characterize cardiovascular modifiable risk ‎factors of AD and determine their contribution in disease development in this country compared ‎to the world.‎

## Materials and Methods

***Search strategy and eligibility criteria***

Medline, Scopus, and Cochrane databases were systematically searched from inception to May 2014 to retrieve systematic reviews and/or meta-analyses focused on the association between AD and seven modifiable risk factors included diabetes, HTN, physical inactivity, smoking, hypercholesterolemia, and overweight and obesity. MeSH terms of (“Alzheimer” OR “dementia) in combination with [“diabetes mellitus (DM)” OR “hyperglycemia” OR “smoking” OR “HTN” OR “physical activity” OR “physical inactivity” OR “exercise” OR “overweight”, OR “obesity”] were searched in mentioned databases. The types of studies were restricted to “systematic reviews” in Medline (PubMed) and to “reviews” in Scopus. Moreover, references of selected studies were evaluated for additional relevant studies. Only papers in English were included in the study. Two authors (NH and LS) reviewed titles and abstracts of articles independently to select more potentially relevant studies and third person (ZR) were consulted if any disagreement took place. 

***Inclusion and exclusion criteria***

Inclusion criteria: (a) systematic review and/or meta-analysis; (b) addressing the association between diabetes, HTN, physical inactivity, smoking, hypercholesterolemia, and overweight or obesity with AD.

Exclusion criteria: (a) other types of studies; (b) assessment of cognitive decline, other types of dementia or dementia as a whole instead of AD.

***Quality assessment***

The quality of relevant studies according to their titles, abstracts, and inclusion and exclusion criteria was evaluated by two authors (NH and LS) independently using “A Measurement Tool to Assess Systematic Reviews” (AMSTAR) as a critical appraisal checklist for quality assessment of systematic reviews and meta-analysis.^11^ There were 11 questions with four types of answer (yes, no, cannot answer, not applicable) in AMSTAR and only questions with “yes” answer had 1+ score. In this manner, papers rated as low (0-4), medium (5-8), and high (9-11) quality according to their scores.^12^ Finally, medium and high-quality studies were selected for data extraction. 

***Data extraction***

Two authors (LS and ZR) independently reviewed selected studies to extract the following information: first author’s name, publication year, searched databases, types of studies, risk factor (exposure), doing meta-analysis and effect size (if provided) and finally conflict of interest. Any conflict between two authors resolved with consult of third person (NH). 

***Population attributable fraction (PAF)***

The PAF is defined as the proportion of patients with specific disease attributed to a risk factor, and subsequently, this proportion could be avoided in terms of risk factor elimination.^13^ With respect to the Levin’s formula,^13^ calculation of PAF would be done with risk factor prevalence (P_RF_) and relative risk (RR) for AD for each risk factor as it is shown in the following formula:


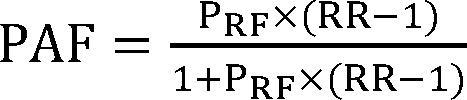


We applied adjusted estimates of risk factors to this formula. RRs or estimates of them [odds ratio (OR)] were obtained from meta-analyses. If there were more than one study on a specific risk factor, the study with higher quality was selected. In the case of assessing smoking, because tobacco industries usually support the studies focused on tobacco (which could affect the outcomes), we selected the meta-analyses considering tobacco industry affiliation. We acquired most recent reported prevalence for each risk factor among Iranian and the world population through simple search in the literature. If reported risk was restricted to a specific age group, we restricted estimates of prevalence to that age group.^14^^-^^17^ We reported 95% confidence interval (CI) for RR and also we calculated 95% CI for PAF using lower and upper limit of selected RR of each risk factor. Finally, combined PAF was calculated using the following formula:


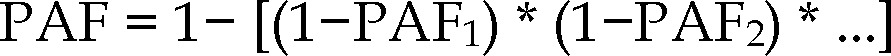


All data organization and processing were performed using Endnote and Excel 2010 software.

## Results

***Study selection***

**Figure 1:**
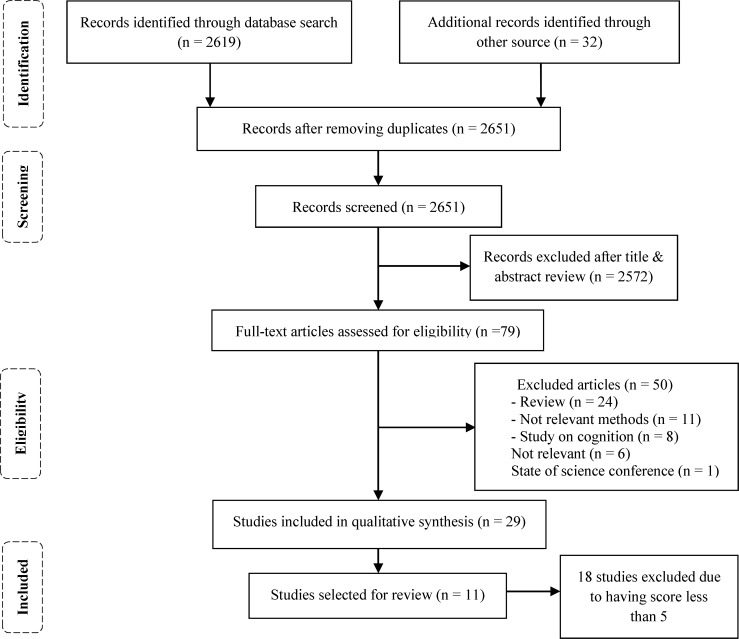
Flow chart of selected studies to review

After conducting systematic search, 2651 articles were identified. We selected 79 potentially relevant articles according to titles and abstracts. Among these, 29 studies were selected based on the inclusion/exclusion criteria (Figure 1). 11 studies with a score of five or more were eligible for data extraction.^12^ Characteristics of the eligible studies are displayed in table 1.

**Table 1:** Characteristics of systematic reviews describing the relationship between seven modifiable risk factors and Alzheimer

**Review name**	**Searched databases**	**Type of studies included**	**Number of studies included**	**Total number of participants**	**Sex**	**Age (year)**	**Exposure (risk factor)**	**Time of follow-up**	**Quality of study**	**Effect size (CI) for AD**	**Conflict of interest**	**Meta-analysis (+/−)**
Stern and Konno^24^	Medline, CINAHL, Embase, Cochrane (Central), PsycINFO, PubMed, EBM Reviews, ISI, ERIC, Austhealth Health Scopus	Case-control	2	18,024-55,136 for case-control		≥ 60	Physical leisure activities	6 study for 5-10 year, 1 study for 5 years	5	-	None	−
		Cohort	15	29435-461534 for cohort								
								5 study for < 5 years, for midlife study 21-36 years				
Lu et al.^18^	PubMed, PsycINFO	Prospective cohort	21 (8 on AD)	23,257 (for AD, vascular and all cause dementia)		69-83	DM	2.1 years to more than 10 years	5	1.39 (1.16-1.66)	None	+
Hamer and Chida^23^	Medline, Cochrane ISI	Prospective cohort	16 (6 on AD)	13771 (in meta for AD)	F/M	In one study 39-64 years with 21 years follow-up and others ≥ 65	Physical inactivity	5-21 years (in meta)	5	0.55 (0.36-0.84)	None	+
Lee et al.^21^	PubMed, Embase, and PsycINFO	Prospective cohort	37	_	_	≥ 65	Physical inactivity, Smoking, BMI, Alchohol, Diet	1.8-20.9 years	5	-	None	−
								2-25 years				
								18-36 years				
								4-24 years				
								3.9-32 years				
Cataldo et al.^20^	PubMed, PsycINFO, Cochrane CENTRAL Google Scholar	Case-control	26	_	_	_	Smoking	1.5-27 years for cohort studies	5	RR: 1.72 (1.33-2.12) (for cohort studies of average quality in 2007), Controlling for study design, quality, secular	No role for sponsor in design, conduct or writing manuscript	+
										Trend and tobacco industry affiliation		
		Cohort	17									
Anstey et al.^22^	PubMed, PsycINFO, Cochrane	Prospective cohort	16 (11 on AD)	Low versus nl BMI in midlife: 8259 Overweight versus nl BMI in midlife : 13506 Obese versus nl BMI in midlife: 9401 Obese versus not obese BMI in late: 4067	F/M	42-74	BMI	29.7 years (BMI in midlife), 6.82 years (BMI in late-life)	6	Low/nl BMI in midlife: RR: 1.96 (1.32-2.92) Overweight versus nl BMI in midlife: RR: 1.35 (1.19-1.54) Obese versus normal BMI in midlife: RR: 2.04 (1.59-2.62) Obese versus not obese BMI in late-life: RR: 1.46 (0.97-2.21)	None	+
Almeida et al.^19^	Medline, PsycINFO	Case-control	21	5323 in case control		-	Smoking	2-27 years for cohorts	6	Case control* OR: 0.82 (0.70-0.97) Four case control** OR: 0.82 (0.53-1.27) Cohort studies RR: 1.10 (0.94-1.29) Two cohort***: RR 1.99 (1.33-2.98)	….	+
		Cohort	8	43885 in cohort								
Anstey et al.^27^	PubMed, PsycINFO, Cochrane CENTRAL	Prospective cohort	18 (8 for TC and AD)	14,331	F/M	40-78	Total cholesterol	4.8-29 (mean: 13.34) years	5	RR: 0.85 (0.67-1.10)† RR: 1.03 (0.79-1.35)†† RR: 0.85 (0.65-1.12)†††	….	+
Power et al.^26^	Medline, Embase, Web of Science, Google Scholar, CINAHL, Review of citations from relevant articles, consultation with experts hand searches of publication archives from cohorts	Prospective cohort, Nested case-control	18			> 50	HTN	3.2-27 years	5	RR: 0.98 (0.80-1.19)# RR: 0.95 (0.91-1.00)## RR: 0.94 (85-1.04)###	….	+
Guan et al.^25^	Embase, Medline	Cohort	12	7270 with HTN 8022 without HTN	F/M	> 40	HTN	1-32 years	6	RR: 1.01 (0.87-1.18) in random-effects RR: 1.02 (0.91-1.14) in ﬁxed-effects models	None	+
Cheng et al.^1^	Embase, Medline	Cohort	19 (16 on AD)	5700 with and 36 191 without diabetes	F/M	> 50	DM	1-12.7 years	7	RR: 1.46 (1.20-1.77) in random effect and RR: 1.54 (1.40-1.70) in fixed effect model	….	+

AD: Alzheimer’s disease; BP: Blood pressure; OR: Odds ratio; RR: Relative risk; HTN: Hypertension; BMI: Body mass index; DM: Diabetes mellitus; EBM: Evidence based medicine; ERIC: Education Resources Information Center; CI: Confidence intervals

* Analysis incorporating ORs adjusted for confounding variables (such as age, sex, schooling and alcohol use),

** Analysis that included only the four case-control studies that used matched design,

*** Restricting the analysis to the two cohort studies that described the number of subjects who were smokers at baseline and later developed AD.

† Association between total cholesterol measured in late life and AD between first quartile and second,

†† Association between total cholesterol measured in late life and AD between first quartile and third,

††† Association between total cholesterol measured in late life and AD between first quartile and fourth,

# 10 studies reporting on association between a “history of HTN” and AD,

## 4 studies considering the association between a 10 mm Hg increment in systolic BP and AD,

### 4 studies considering the association between a 10 mm Hg increment in diastolic BP and AD.

***Alzheimer risk factors***

DM Type 2: From selected articles, two had systematically reviewed the association between DM and AD.^1^^,^^18^ Lu et al.^18^ had found 21 prospective cohorts on AD, vascular and all causes of dementia in which eight had addressed the DM and AD association. After data pooling with meta-analysis, they concluded that patients with DM have a higher risk for AD in comparison with non-diabetics [RR = 1.39 (95% CI: 1.17-1.66). Another study with higher quality^1^ that was a meta-analysis on 16 prospective cohorts with 41891 participants, had revealed that diabetic patients were 1.46 times more risky than patients without diabetes for AD [RR = 1.46 (95% CI: 1.20-1.77)]. We used RR of 1.46 for estimation of relevant PAF.

Smoking: There were three systematic reviews that evaluated the association between smoking and AD.^19^^-^^21^ One of them^21^ discussed multiple modifiable risk factors’ effects on various types of cognitive impairment. They did not do meta-analysis because of heterogeneity of data. Other two studies had conducted meta-analysis. Cataldo et al.,^20^ had identified 26 case control and 17 cohort studies and had included them in meta-analysis according to their designs and existence of tobacco industry affiliation. At the end, they had reported multiple pooled RRs. The best one was RR: 1.72 (1.33-2.12) that was based on cohort studies without tobacco industry affiliation, published in 2007. This study had a medium quality in which possible confounders such as tobacco industry affiliations were controlled. Another meta-analysis was conducted on 21 case control and 8 cohorts studies by Almeida et al.^19^ After controlling various confounders, two ORs for case controls and two RRs for cohorts were measured that had a conflict with each other. For two cohorts, in which participants were current smokers at the beginning of the follow-up, RR was 1.99 (1.33-2.98). We used RR of 1.72 (1.33-2.12) to calculate PAF for AD.

Overweight and Obesity: Two studies addressed the relationship between body mass index (BMI) and AD.^21^^,^^22^ There was no meta-analysis in the study of Lee et al.^21^ However, other had run meta-analysis on 11 prospective cohorts to illustrate any association between BMI and AD.^22^ There were significant associations between both overweight and obesity in middle age and incidence of AD later in life [RR: 1.35 (1.19-1.54) and RR: 2.04 (1.59-2.62), respectively].

Physical inactivity: Three out of 11 selected studies had addressed association between physical activity and AD.^21^^,^^23^^,^^24^ Of these, two studies could not perform meta-analysis due to the existence of heterogeneity and their final results were explained just in narrative form.^21^^,^^24^ There was just one meta-analysis on 6 longitudinal studies that had tried to investigate the association between physical activity and AD.^23^ The pooled RR was 0.55 (0.36-0.84), meaning that physical activity behaves as a protective factor and has the ability to decline incidence of AD in the late life. As in available studies, physical activity instead of inactivity was in the center of scientists’ attention and it played a protective role in AD, we reversed these values to display risk of physical inactivity (RR: 1.81, 1.19-2.77).

HTN: In 2011, two groups of medical scientists separately conducted comprehensive systematic reviews and meta-analysis on the association between HTN and AD.^25^^,^^26^ One of them pooled data using both fixed and random effect models. Another one considered the impact of an increase in systolic blood pressure (BP), diastolic BP (DBP), history of HTN, and presence of HTN on AD at the beginning of the study. None of them could find any significant association as mentioned in table 1.

Hypercholesterolemia: Our search could find just one meta-analysis assessing the impact of high blood cholesterol level on AD.^27^ The authors concluded that high level of cholesterol had a significant effect on AD neither in midlife, nor in late life. 

***Risk factors prevalence***

We searched the prevalence of the each risk factor among Iranian and world population in the literature. Ultimately, we decided to use two reports from the World Health Organization (WHO) to extract the prevalence of smoking, diabetes, and physical inactivity in Iran^15^ and the world.^14^ Because overweight and obesity had a positive relationship with AD just in middle age, we could calculate midlife overweight and obesity using the size of population and prevalence of overweight and obesity in 40-60 years old population.^22^ Hence, we used one population-based study that had provided prevalence in favored age groups for Iran^16^ and another one for the world.^17^

**Table 2:** Population attributable fraction (PAF) for Iran and the world

**Risk factor**	**Prevalence (%)**	**RR**	**PAF**
Iran			
DM	8.3	1.46 (1.20-1.77)	0.036 (0.016-0.060)
Smoking	8.1	1.72 (1.33-2.12)	0.055 (0.026-0.083)
Physical inactivity	35.7	1.81 (1.19-2.77)	0.220 (0.060-0.380)
Midlife overweight	3.3	1.35 (1.19-1.54)	0.011 (0.006-0.017)
Midlife obesity	1.6	2.04 (1.59-2.62)	0.016 (0.009-0.025)
World			
DM	11.0	1.46 (1.20-1.77)	0.048 (0.021-0.078)
Smoking	26.0	1.72 (1.33-2.12)	0.157 (0.079-0.225)
Physical inactivity	17.0	1.81 (1.19-2.77)	0.120 (0.030-0.230)
Midlife overweight	5.8	1.35 (1.19-1.54)	0.020 (0.010-0.030)
Midlife obesity	2.5	2.04 (1.59-2.62)	0.025 (0.014-0.039)

PAF: Population attributable fraction; DM: Diabetes mellitus; RR: Relative risk

Unfortunately, because there was no population-based study for Iran that could provide overweight and obesity in 40-60 years old population, we used 45-65 years old group as midlife.^28^ The sizes of desired age groups of the population for both Iran and the world were derived from the website of the United Nations, Department of Economic and Social Affairs.^29^

***PAF***

PAFs calculated for the targeted risk factors for Iran and the world are presented in table 2. In Iran, physical inactivity and smoking had the first degree in PAF for AD with 22 and 5%, respectively. The calculated PAF for the world was different. Smoking and physical inactivity had the largest PAF (15.7 and 12%, respectively) for the world. It means that more than one-quarter of AD in Iran and the world was attributed to two modifiable risk factors. Combined PAF for all risk factors were 0.308 and 0.352 for Iran and the world, respectively..

## Discussion

The aim of this study was to determine any association between seven potentially modifiable risk factors and AD. Finally, we estimated PAF for five risk factors except HTN and hypercholesterolemia. 

Based on two high-quality meta-analyses,^19^^,^^20^ there was an association between smoking and increased the risk of AD. Smoking had the highest PAF in world and was in the second order for Iran among five assessed risk factors. It has been shown that metabolites derived from increasing arachidonic acid peroxidation in smokers’ plasma cause oxidative stress.^30^ Furthermore, a high level of damage could be observed in the cerebral cortex of smokers due to the presence of free radicals.^31^ As a fact, free radicals can kill neurons or alter their function, leading to AD.^32^ Moreover, smoking could induce atherothrombosis through several mechanisms like inflammation, proliferation of smooth muscles and platelet and leukocyte activation,^33^ which can play a role in AD.^34^

Besides, the results of two systematic reviews and meta-analyses have mentioned that diabetic patients are at higher risk for AD than non-diabetics.^1^^,^^18^ Both diabetes and AD are common and debilitating diseases particularly in the elderly.^3^ Diabetes contributes to AD development not only as an interactive factor with the other cardiovascular and neurodestructive risk factors such as HTN and dyslipidemia but also as an independent risk factor.^35^

Two main mechanisms explain the effect of diabetes on AD development. The first is vascular mechanisms such as microinfarctions, increased blood brain barrier permeability, increased beta-amyloid production, and it’s deposition in the white matter.^4^ In addition, resistance to insulin and hyperinsulinemia as two important components of diabetes can contribute in vascular mechanisms. The second mechanism is the non-vascular one; hyperglycemia that leads to anaerobic metabolism and lactic acidosis, decreases cholinergic transmission through blood brain barrier and causes glutamate receptors dysfunction.^36^^-^^40^

Overweight and obesity in midlife can increase the risk of AD in later life.^22^ One possible explanation of this relationship is that obesity is a risk factor for DM^41^ which is also a risk factor for AD by itself.^1^^,^^18^ Another explanation is that there is a relationship between obesity and resistance to insulin^42^ and insulin resistance could play a role in AD.^43^ With respect to PAF, and despite the importance of these two risk factors, reduction of them has lesser effects on AD prevention comparing to the others, both in Iran and the world.

Among five evaluated risk factors in the current study, physical inactivity had the highest PAF in Iran. Physical inactivity acts both directly and indirectly through obesity and diabetes which are proven risk factors for AD.^41^ On the other hand, there is some trustworthy evidence that physical activity has preventive effects on AD.^23^ Furthermore, it has been shown that exercise could lead to activation of growth factor cascade that is essential for neurogenesis and vascular function. Besides, exercise could lessen some risk factors of metabolic syndrome which contributes to brain dysfunction and degenerative process.^44^ Exercise could assist brain function through boosting antioxidant activity and oxidant destruction renewing system.^45^

The rationale for selection of these risk factors was that these risk factors were potentially modifiable and the existence of any association between them and AD could help policy makers to plan for prevention of this problem. Because of high PAF for physical inactivity in Iran, health policy makers should focus on building and strengthening infrastructure in collaboration with other sectors to promote physical activity as a protective behavior. For this purpose, studies on cost-effectiveness of this intervention should be encouraged. Besides, food market policies should be revised to reduce the risk of DM, obesity, and overweight problems. In addition, screening of people for diabetes and obesity could be offered. Finally, encouraging tobacco cessation especially in young people can have an enormous effect on AD reduction.

The strength of our study is doing a wide and systematic review for finding risk factors of AD and estimating PAF for the first time in Iran. 

However, our study has some limitations. The first one was restriction of our search to just English papers. Second, midlife has several definitions in terms of age span, so for obesity and overweight, we consider age group of 40-60 as midlife. However, because of unavailable data for this age group, calculation was done on 45-65 years old adults. Finally, as a third limitation, although physical activity has different types in terms of intensity and frequency that may have various effects, we considered it as a general protective factor.

## Conclusion

Our findings showed that about one-third of cases of AD were attributed to five modifiable risk factors which are almost similar in both Iran and the world in terms of combined PAF. If we could eliminate physical inactivity and smoking among population, about 27% of AD would be avoided. Therefore, a large number of AD cases could be avoided with lifestyle modification.

## Notes:

**How to cite this article:** Hazar N, Seddigh L, Rampisheh Z, Nojomi M. Population attributable fraction of modifiable risk factors for Alzheimer disease: a systematic review of systematic reviews. Iran J Neurol 2016; 15(3): 164-72.
